# Tethered tertiary amines as solid-state n-type dopants for solution-processable organic semiconductors†
†Electronic supplementary information (ESI) available: Materials and experimental methods, details of characterization experiments (NMR, EPR, XPS, UV/VIS/NIR, FTIR). See DOI: 10.1039/c5sc04217h


**DOI:** 10.1039/c5sc04217h

**Published:** 2015-12-09

**Authors:** Boris Russ, Maxwell J. Robb, Bhooshan C. Popere, Erin E. Perry, Cheng-Kang Mai, Stephanie L. Fronk, Shrayesh N. Patel, Thomas E. Mates, Guillermo C. Bazan, Jeffrey J. Urban, Michael L. Chabinyc, Craig J. Hawker, Rachel A. Segalman

**Affiliations:** a Department of Chemical and Biomolecular Engineering , University of California , Berkeley , CA 94720 , USA; b Lawrence Berkeley National Laboratory , 1 Cyclotron Road , Berkeley , CA 94720 , USA; c Department of Chemistry and Biochemistry , University of California , Santa Barbara , CA 93106 , USA; d Department of Chemical Engineering , University of California , Santa Barbara , CA 93106 , USA . Email: segalman@engineering.ucsb.edu; e Materials Department , University of California , Santa Barbara , CA 93117 , USA . Email: mchabinyc@engineering.ucsb.edu ; Email: hawker@mrl.ucsb.edu

## Abstract

A scarcity of stable n-type doping strategies compatible with facile processing has been a major impediment to the advancement of organic electronic devices. Localizing dopants near the cores of conductive molecules can lead to improved efficacy of doping. We and others recently showed the effectiveness of tethering dopants covalently to an electron-deficient aromatic molecule using trimethylammonium functionalization with hydroxide counterions linked to a perylene diimide core by alkyl spacers. In this work, we demonstrate that, contrary to previous hypotheses, the main driver responsible for the highly effective doping observed in thin films is the formation of tethered tertiary amine moieties during thin film processing. Furthermore, we demonstrate that tethered tertiary amine groups are powerful and general n-doping motifs for the successful generation of free electron carriers in the solid-state, not only when coupled to the perylene diimide molecular core, but also when linked with other small molecule systems including naphthalene diimide, diketopyrrolopyrrole, and fullerene derivatives. Our findings help expand a promising molecular design strategy for future enhancements of n-type organic electronic materials.

## Abstract 2

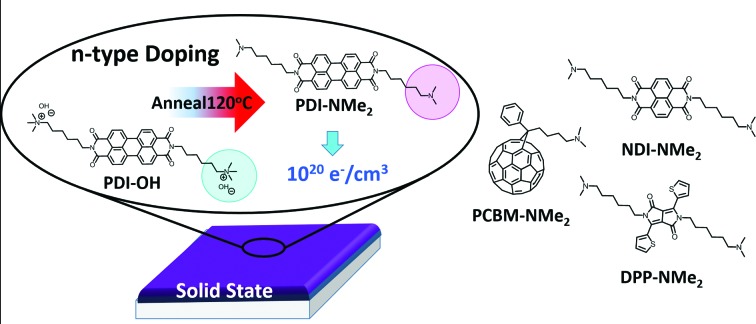
Tertiary amines covalently tethered to electron-deficient aromatic molecules by alkyl spacers enable solid-state n-doping.

## Introduction

The promise of low-cost and scalable electronic devices has attracted great interest in the development of active organic materials for applications, including light emitting devices (OLEDs),^1^–^3^ photovoltaics (OPVs),^4^–^6^ thin film transistors (TFTs),^7^–^9^ and thermoelectrics.^10^–^13^ Key to realizing the potential of such organic electronics are controlled hole (p-type) and electron (n-type) doping strategies. Doping can enable improvement in devices by: (1) boosting effective carrier mobilities by filling trap states in TFTs, (2) tuning electrical conductivity, and (3) aligning energy levels at active material interfaces and metal charge injection/extraction contacts to reduce device operating voltages in OLEDs or to optimize open circuit voltages in OPVs. In thermoelectrics, the carrier concentration must be controlled to optimize the thermoelectric figure of merit that depends on the electrical conductivity, Seebeck coefficient, and thermal conductivity.

While strategies for p-type doping have advanced significantly,^10^,^14^,^15^ the low electron affinities of organic semiconductors (3 to 4 eV) have limited the options for n-doping of materials.^15^ To enable scalable and cost-effective fabrication approaches, doping strategies compatible with solution-processing are desired. Extrinsic dopants, such as cleavable dimeric organometallic complexes,^16^ hydride donors, like N-DMBI,^7^,^17^ and charged tetrabutylammonium salts,^18^ in which counterions have been proposed to act as sacrificial electron donors, are all promising approaches.^16^ Molecular design is also important to prevent phase segregation of dopants and the semiconducting material.^19^–^21^ We and others recently demonstrated an alternative strategy to using extrinsic dopants that simultaneously enables solution-processability and localizes the dopants within the active host matrix to create high performing thermoelectric materials.^12^,^22^,^23^ Specifically, quaternary ammonium cations with hydroxide counterions were tethered to perylene diimide (PDI) using alkyl spacers. Doping of these water-soluble charged PDIs was accomplished through drying and a low temperature thermal treatment of cast films, resulting in exceptional charge carrier densities in the solid-state (10^20^ to 10^21^ carriers per cm^3^).^12^

**Fig. 1:**
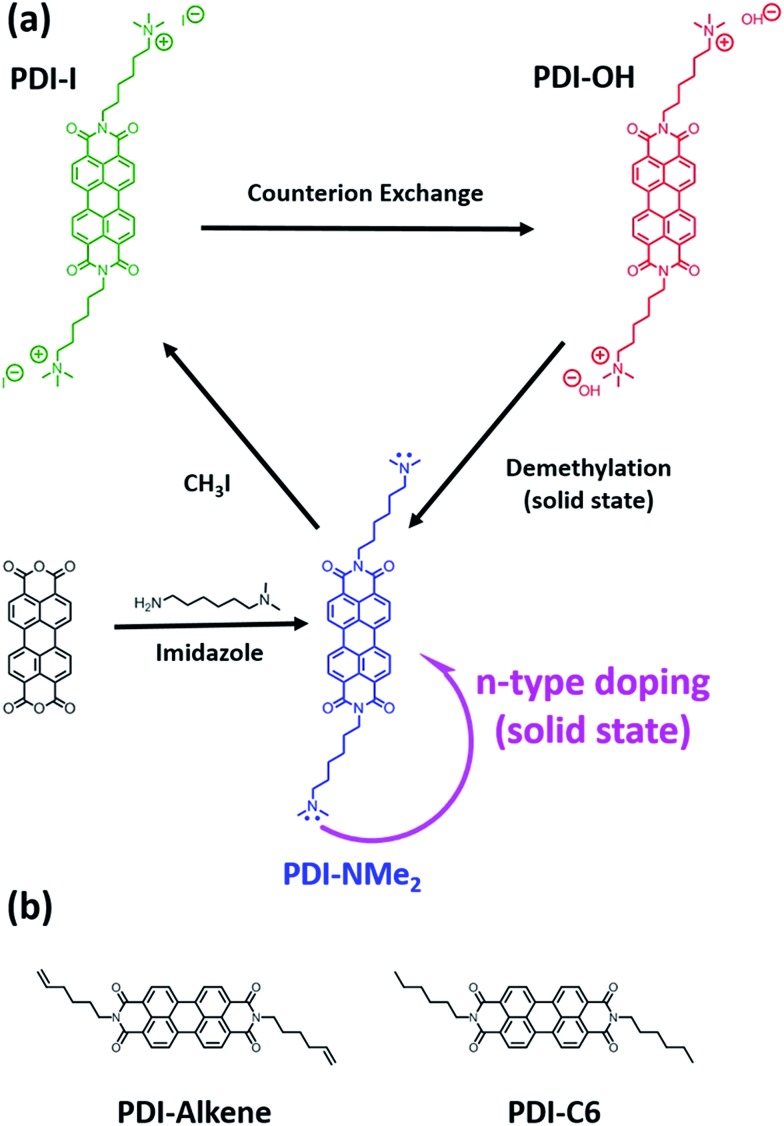
Chemical transformation of side-chain functionality precedes n-type doping in water-soluble perylene diimides (PDIs). (a) Quaternary functionalization, used to render the PDI core water-soluble (PDI-I), is achieved through methylation of PDI-NMe_2_. When hydroxide anions are used as the counteranions (PDI-OH), charge transfer promoting tertiary amines are recovered upon solid-state thermal treatment by demethylation of the quaternary ammonium groups. Tertiary amine functional groups (in PDI-NMe_2_) induce charge transfer in solid-state under mild thermal treatment. (b) Structures of PDI controls evaluated in this study.

Despite the demonstrated performance of these materials, the underlying mechanism for the self-doping phenomenon was not well understood. It was initially proposed that doping could result from counteranions participating in a partial electron transfer during molecular compaction with the cationic functionalities remaining inert.^23^ However, tetraalkylammonium functional groups are known to react with hydroxide ions, leaving some uncertainty about the identity of the active doping species in the solid-state.^24^ To aid the design of future self-doping materials, we sought to identify the active motif responsible for n-type doping and understand its role in the doping mechanism. In this work, we demonstrate that the hydroxide counterions and the alkyl tethered trimethylammonium functional groups in the PDI system are chemical precursors in the doping process and react in the solid-state to produce alkyl tethered dimethylamino end groups (Fig. 1a). It is these resulting tertiary amines that are involved in the electron transfer observed in thin films. Furthermore, we show that solid-state doping using tethered tertiary amine functional groups can be extended to other n-type small molecule systems, including naphthalene diimide (NDI), diketopyrrolopyrrole (DPP), and fullerene derivatives. The combination of streamlined synthesis and the generality of the localized doping approach presents a new design paradigm with major implications in the development of solution-processable n-type organic materials highly relevant to the broad organic electronic materials community.

## Results and discussion

Initially, a series of functionalized PDI derivatives was synthesized (Fig. 1a) and fully characterized (see ESI†). Briefly, bis(6-dimethylaminohexyl) functionalized PDI (PDI-NMe_2_) was synthesized in a one-step reaction starting from perylene tetracarboxylic acid dianhydride.^12^,^25^ To form the alkyl tethered trimethylammonium functionalized PDI with hydroxide counterions, PDI-NMe_2_ was first methylated using CH_3_I (forming PDI-I) and the I^–^ anion subsequently exchanged for OH^–^ (resulting in PDI-OH). Additional PDI variants lacking the dimethylamino group (Fig. 1b), namely PDI-C6 (*N*-hexyl) and PDI-alkene (*N*-hex-5-ene substituents, see ESI† for synthesis), were used as controls in this study.

Given the inherent instability of tetraalkylammonium salts,^26^–^28^ we hypothesized that several possible chemical reactions might be involved in the self-doping of charged PDIs in thin films, primarily *via* transformation of the quaternary ammonium groups during processing. For example, Hofmann elimination involving abstraction of the β-hydrogens by the hydroxide would result in elimination of trimethylamine, producing water and a terminal alkene functional group (PDI-alkene).^24^ Alternatively, nucleophilic attack of a methyl group by the hydroxide would liberate methanol resulting in neutral dimethylamino functionalities tethered by alkyl chains to the PDI core (PDI-NMe_2_).^26^ The latter substitution pathway seemed most plausible in the doping process. Firstly, hydroxide anions are known to be strong nucleophiles in non-aqueous media^29^ and have recently been shown to drive chemical transformations *via* nucleophilic attack leading to n-doping intermediates in fullerene species.^30^ Secondly, it is well established that tertiary amines can participate in electron transfer reactions with acceptors. Studies of photoinduced electron transfer between amine-containing species and sensitizers (dyes) in solution have been a topic of research for decades.^31^–^35^ In recent work, for example, the combination of triethylamine and PDI was shown to be an effective photoredox catalyst system for the reduction of aryl halides.^36^ The presence of amines has also been implicated in solid-state electron transfer, from control of contact work-functions^37^–^39^ to induction of photochromic and photomechanical responses.^40^

**Fig. 2:**
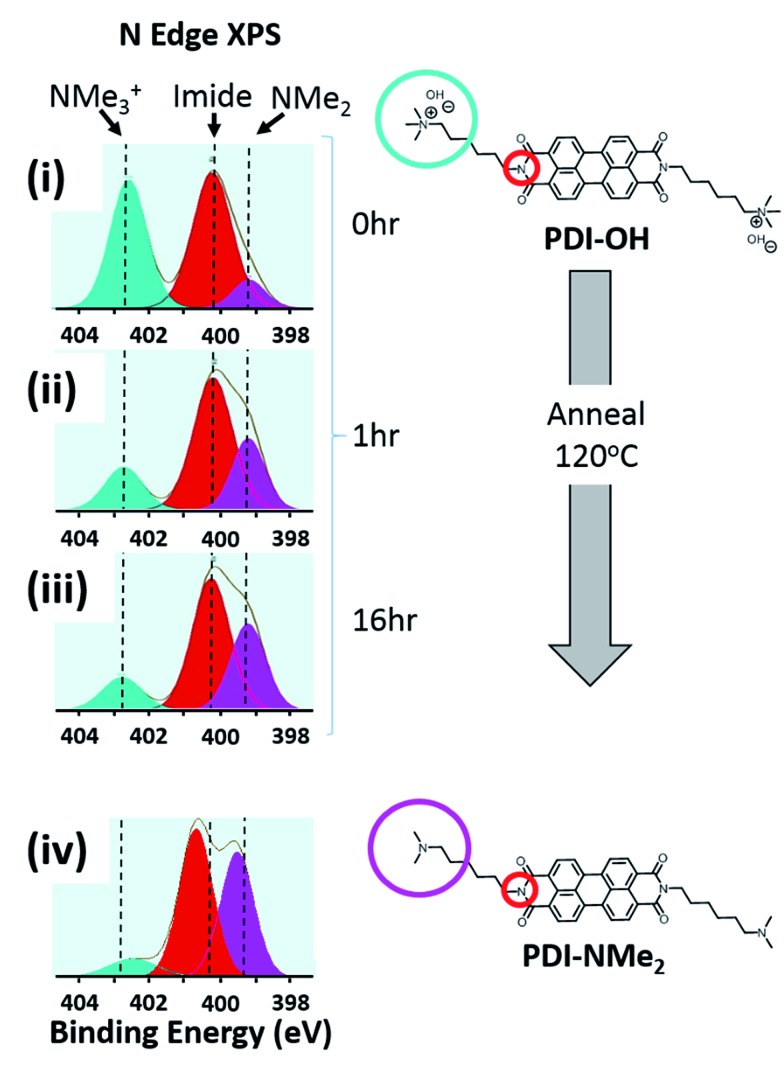
Conversion of NMe_3_^+^ to NMe_2_ in PDI-OH proceeds upon thin film annealing at 120 °C ((i) 0 h, (ii) 1 h, (iii) 16 h) as observed *via* XPS (N 1s spectra). As expected, after extended annealing, the XPS spectrum of films of PDI-OH closely resembles that of PDI-NMe_2_ (iv).

Changes in the local nitrogen environment of the functional groups, as tracked with X-ray photoelectron spectroscopy (XPS), suggest that conversion of quaternary ammonium cations to tertiary amines does take place upon thin film formation and annealing. As shown in Fig. 2, the fraction of quaternary ammonium functionalization (cyan shading) diminishes upon drying and annealing of the PDI-OH films, while the concentration of tertiary amines increases (pink shading). For comparison, the signal fraction corresponding to the PDI imide nitrogen atoms (shaded red) remains unchanged (∼50%, as expected). Upon film drying and prior to annealing (i), tertiary amines already compose ∼15% of all functional groups (see Fig. S1† for quantitative details). Annealing thin films of PDI-OH at 120 °C (ii, iii) propagates end group conversion; after 16 hours, the annealed PDI-OH films closely resemble annealed films of PDI-NMe_2_ (iv), with only minor amounts of ammonium signal still remaining. In contrast, PDI-I (PDI-OH precursor with I^–^ counterions instead of OH^–^) thin films did not display noticeable tertiary amine signal even after extended annealing (not shown), supporting the presumption that the chemical transformation is strongly dependent on the nucleophilicity of the hydroxide anion. A decrease of the broad absorption peak in the FTIR spectra in the region characteristic of hydroxyl groups between 2000–4000 cm^–1^ is also observed upon annealing PDI-OH films (Fig. S2†). These observations are further supported by MALDI-MS measurements that demonstrate the loss of a methyl group upon annealing of PDI-OH. The main signal with *m*/*z* = 674, corresponding to the symmetric PDI with NMe_3_^+^ dications, disappears after annealing with the concomitant enrichment of a signal at *m*/*z* = 659, which corresponds to the unsymmetrical PDI containing one tertiary amine and one quaternary amine group (Fig. S3†). Only a small peak of the symmetric PDI with two alkyl tethered dimethylamino endgroups (*m*/*z* = 645) is seen.

**Fig. 3:**
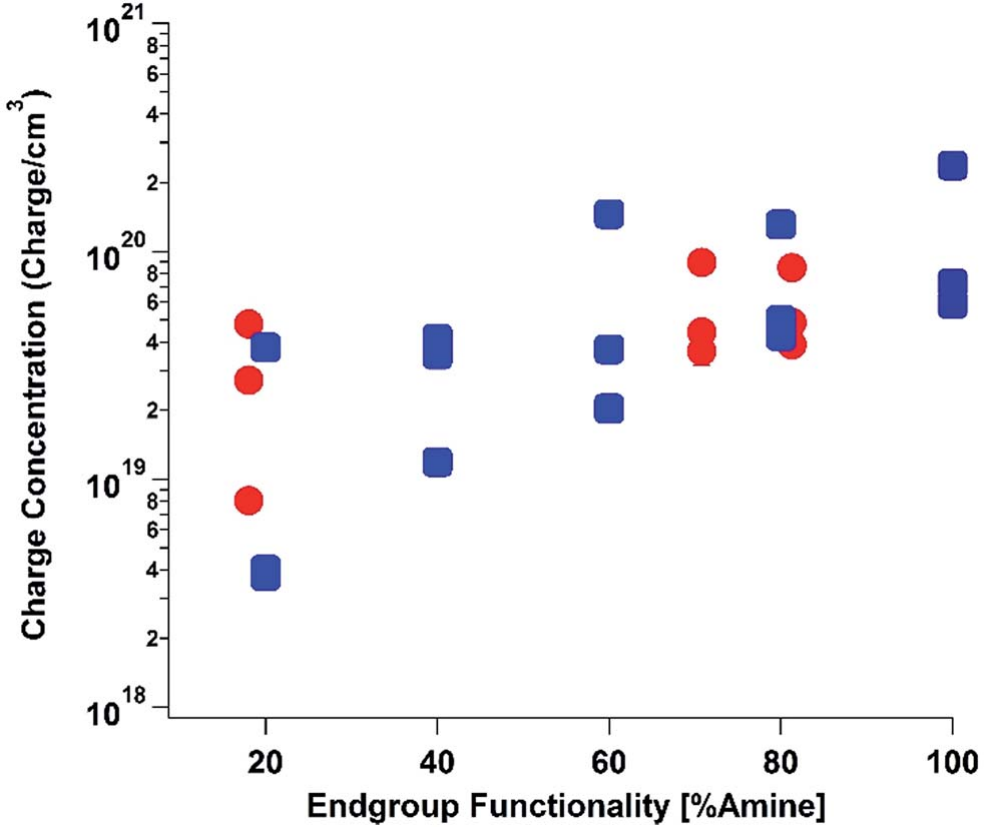
Electron paramagnetic resonance (EPR). Quantitative measurements of charge carrier concentration in PDI samples were done in triplicate for all samples: (1) PDI-NMe_2_/PDI-C6 composites with varying PDI-NMe_2_ content (blue squares), (2) annealed PDI-OH samples (red circles). Error bars for each measurement are on the scale of the data points.

**Fig. 4:**
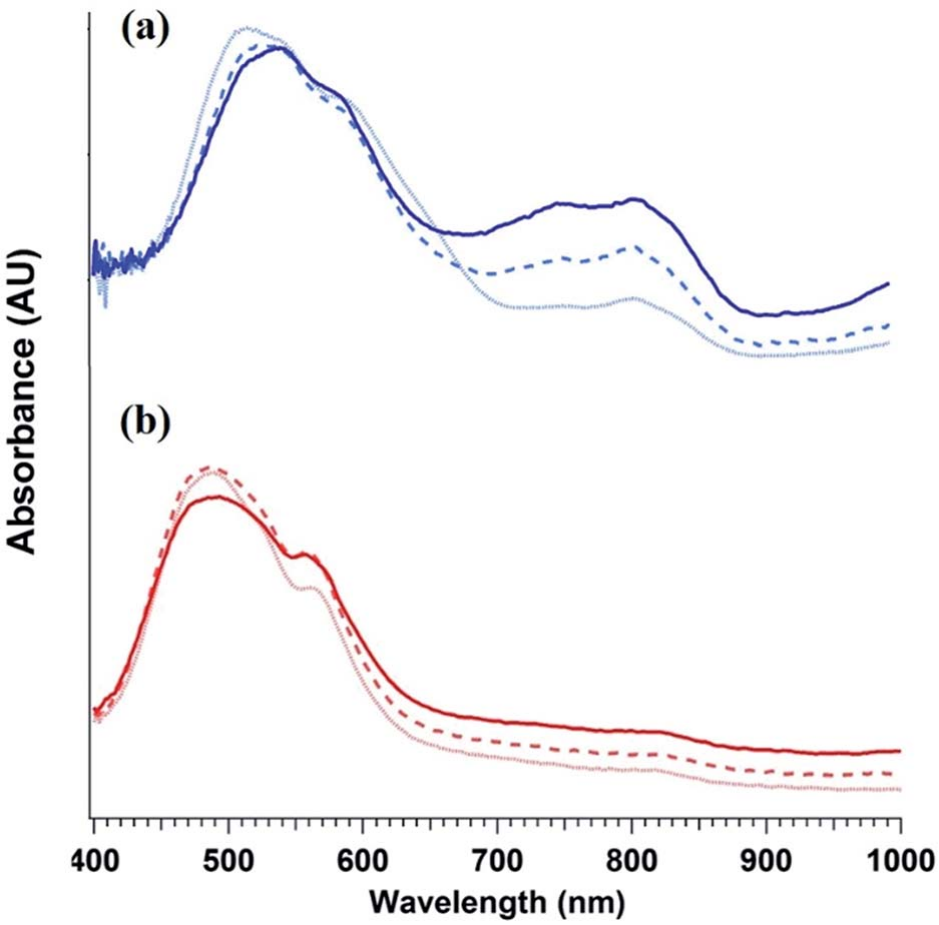
Evolution PDI thin film optical absorption spectra with anneal time: 0 min (light dotted line), 20 min (dashed line), and 1 h (dark solid line) for (a) PDI-OH and (b) PDI-NMe_2_.

To elucidate the ability of trialkylamines to form charge carriers in the solid state from both the alkyl tethered tertiary amines liberated in annealed PDI-OH and also in PDI-NMe_2_, we used quantitative electron paramagnetic resonance (EPR) spectroscopy (Fig. 3 and S4†). Control samples, PDI-C6 and PDI-alkene, showed negligible charge concentration. In PDI-NMe_2_/PDI-C6 composite films, with PDI-C6 content varied to adjust the overall fraction of amine functional groups, the polaron densities are large (∼10^20^ carriers per cm^3^) and roughly scale with the fraction of amine functional groups. The charge concentrations for annealed PDI-OH thin films, in which the fraction of amine functional groups was estimated from the XPS data, fall in line with the established tertiary amine dependence. Distinct radical anion (polaron) related features (peaks at ∼730 nm, 800 nm, and 1000 nm) in the UV/VIS/NIR spectra of PDI-OH thin films, seen in Fig. 4, are consistent with the EPR findings. Upon annealing, the intensity of the polaron bands for PDI-OH and PDI-NMe_2_ thin films increases, while no changes are observed in films of PDI-I and PDI-C6 controls (Fig. S5†). It is noted that the polaron features are more pronounced with PDI-OH than with PDI-NMe_2_ in the UV/VIS/NIR spectra (relative to the absorption intensity of the neutral PDI features in the visible range). The relative difference in polaron intensities is also seen in FTIR comparing the asymmetry in the absorption feature at ∼1700 cm^–1^ (corresponding to the carbonyl group) relative to the peak at 1650 cm^–1^ with a greater asymmetry of the peaks suggesting stronger doping (Fig. S2†).^41^ We suspect that the polaron signatures appear weaker for the PDI-NMe_2_ sample because of air exposure during the optical spectroscopy measurements^36^ (in contrast, EPR and XPS measurements were collected under nitrogen and vacuum environments, respectively). This suggests that residual cationic ammonium functionality may play an important role in providing enhanced air stability to samples of annealed PDI-OH.

As the characteristic polaron features are not observed in solutions of either PDI-OH or PDI-NMe_2_ (Fig. S6†), electron transfer (ET) is likely to occur in the solid state. Furthermore, simply casting the PDI-NMe_2_ films at 30 °C without additional thermal treatment was sufficient to highly dope the material as confirmed by EPR (Fig. S4†). Given that the ionization potential of a tertiary amine and the electron affinity of the PDI are separated by ∼2.6 eV, it is intriguing that the ET appears to be spontaneous. Coulombic stabilization of the proximal radical anion and cation and polarization in the solid can help diminish the overall energetic barrier for charge transfer, but even accounting for these factors, some external excitation source must be helping overcome the remaining energy barrier in excess of 1 eV. Despite efforts to preclude exposure of the films to light during processing, doping was still observed in PDI-NMe_2_ films; however, rapid photo-induced excitation during sample transfer cannot be completely ruled out, possibly assisting in the charge generation process (Fig. S8†).^36^ UV light photo-induced ET involving tethered amine functionalities in the solid-state has recently been reported.^40^

An important question to consider is why the observed anion formation is stable, *i.e.* what prevents recombination of any photogenerated charges. It is possible that PDI˙^–^/–NMe_2_˙^+^ radical ion pairs may be formed during the doping process, but given the thermodynamic instability of such amine intermediates, these byproducts may be transient and likely further react to form more stable cationic species. The possibility of protonation of the tertiary amine during this process may explain the small high binding energy peak seen in the XPS for PDI-NMe_2_ samples (Fig. 2iv and S7†). We note that in quantifying the charge carrier concentration, all signal is attributed to the PDI anion radicals given the likely instability of complementary tertiary amine cation radicals formed. The possibility remains that signal from cation radicals could be convoluted in the quantified data, however, even if this was the case, the reported trends in polaron concentrations in the PDI films would be unaffected due to charge neutrality. Solution proton NMR of PDI-NMe_2_ samples before and after the thermal processing (Fig. S9†) were different, suggesting that longterm annealing likely drives some chemical transformations and renders the doping process irreversible. Solid state NMR and transient spectroscopic experiments, which are beyond the scope of this study, should be helpful in identifying the nature of possible intermediates.

**Fig. 5:**
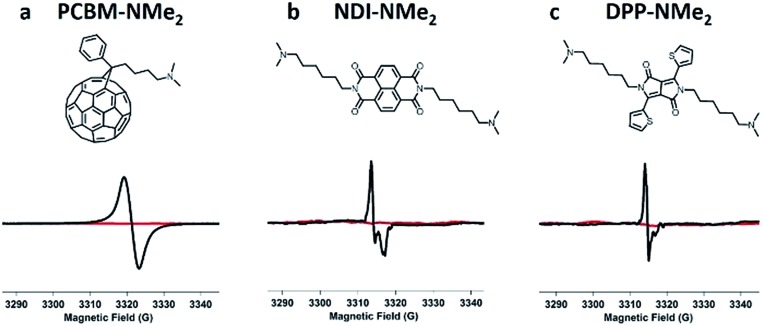
Tethered tertiary amines represent a general design motif for solid-state n-type dopants. Distinct EPR radical signatures are observed for functionalized samples (in black) in three common n-type molecular systems: (a) naphthalene diimide (NDI-NMe_2_), (b) diketopyrrolopyrrole (DPP-NMe_2_), and (c) fullerene (PCBM-NMe_2_) derivatives. EPR signal corresponding to control samples with the same molecular cores but without tertiary amine functionalization are shown in red (see ESI† for structures).

Having identified tethered tertiary amine functional groups as the active motif responsible for low temperature self-doping, we sought to determine if aromatic cores beyond PDIs would undergo electron transfer processes. Thus, dimethylamino functionalized variants (Fig. 5) of naphthalene diimide (NDI-NMe_2_), diketopyrrolopyrrole (DPP-NMe_2_), and fullerene (PCBM-NMe_2_) were synthesized and fully characterized (see ESI†). In a testament to the generality of the doping capability, dimethylamino functionalization successfully led to doping in all three of these molecular systems with LUMO levels between 3.5 and 4.1 eV (Fig. S10†) under mild processing conditions, exemplified by the distinct radical signatures observed in EPR compared to corresponding controls without dimethylamino functionalities (Fig. 5; see ESI† for controls). Because of the generality, this work suggests that solid state electron transfer is likely to occur in many systems beyond PDIs and should be an avenue for exploration to study doping in n-type organic electronic materials. For example, there are many reports of interlayers in solution processed photovoltaics where such processes may have already been observed.^42^–^45^

## Conclusions

In conclusion, we demonstrate that tethered dimethylamino end groups are versatile stand-alone n-doping functionalities for the successful generation of free electron carriers in solid-state films. The generality of this behavior is demonstrated not only when coupled to a perylene diimide molecular core, but also when linked to naphthalene diimide, diketopyrrolopyrrole, and fullerene derivatives. Furthermore, the use of charged quaternary ammonium groups can enable water solubility, while still capitalizing on doping *via* tertiary amines. We show that the hydroxide anion and alkyl tethered trimethylammonium functionality in water-processable charged PDI-OH react in the solid-state to produce alkyl tethered dimethylamino end groups that are responsible for the observed n-type doping. Residual cationic groups in dehydrated films may help stabilize generated charge, thereby adding air stability to the doped films. It should be noted that the reactivity of tetraalkylammonium complexes highlighted in this study may also help shed new light on alternate doping processes involving charged tetraalkylammonium extrinsic dopants. The mechanistic understanding provided here broadens the possibilities for the design of solution-processable n-type organic materials of great interest to the organic electronics community as a whole.

## Author contributions

G. C. B., J. J. U., M. L. C., C. J. H., and R. A. S. conceptualized and guided the experiment. B. R., M. J. R., B. P., C.-K. M., S. F., G. C. B., J. J. U., M. L. C., C. J. H., and R. A. S. designed the molecules. M. J. R., B. P., C.-K. M., S. F. synthesized material and performed an analytical workup of these materials. E. E. P. and C.-K. M. performed EPR sample preparation and measurement and B. R. and E. E. P. performed EPR data analysis. S. N. P. and T. E. M. performed XPS sample preparation and measurement, and B. R. and T. E. M. performed XPS data analysis. B. R. performed optical characterization of the materials. All authors participated in discussion of scientific ideas, and B. R. wrote the manuscript.

## Supplementary Material

Supplementary information
