# Supplementary material for: Tethered tertiary amines as solid-state n-type dopants for solution-processable organic semiconductors
Source: Chem Sci. 2015 Dec 9;7(3):1914–9. doi: 10.1039/c5sc04217h (PMC5966797; doi:10.1039/c5sc04217h)
Supplement: Supplementary file 1 [file SC-007-C5SC04217H-s001.pdf]

Electronic Supplementary Material (ESI) for Chemical Science.
This journal is © The Royal Society of Chemistry 2016

Supporting Information

Tethered tertiary amines as solid-state n-type dopants for solution-processable organic semiconductors

Boris Russ, Maxwell J. Robb, Bhooshan C. Popere, Erin E. Perry, Cheng-Kang Mai, Stephanie L. Fronk, Shrayesh N. Patel, Thomas E. Mates, Guillermo C. Bazan, Jeffrey J. Urban, Michael L. Chabinyc,* Craig J. Hawker,* and Rachel A. Segalman*

Contents

1. MATERIALS AND METHODS S1
2. SYNTHESIS S3
3. FIGURES AND TABLES S10
4. REFERENCES S18

1. MATERIALS AND METHODS

All reagents from commercial sources were used without further purification unless otherwise stated. N,N′-(1-hexyl)-1,4,5,8-naphthalenetetracarboxydiimide (NDI-Control), and 3,6-bis(5-bromo-2-thienyl)-2,5-bis(2-hexyldecyl)-2,5-dihydro-pyrrolo[3,4-c]pyrrole-1,4-dione (DPP-Control) were purchased from Aldrich. [6,6]-Phenyl C61 butyric acid methyl ester (PCBM-Control) was purchased from Solenne. 6-(Dimethylamino)hexylamine was purchased from Matrix Scientific. Deuterated solvents were obtained from Cambridge Isotope Laboratories, Inc.

NMR spectra were recorded using a Varian 500 or 600 MHz spectrometer. All ^{1}H NMR experiments are reported in δ units, parts per million (ppm), and were measured relative to the signal for residual chloroform (7.26 ppm) in deuterated solvent. All ^{13}C NMR spectra were measured in deuterated solvents and are reported in ppm relative to the signals for residual chloroform (77.16 ppm) or 1,1,2,2-tetrachloroethane (73.78 ppm). Mass spectrometry was performed on a Micromass QTOF2 quadrupole/time-of-flight tandem mass spectrometer (ESI) or a Waters GCT Premier time-of-flight mass spectrometer (EI). MALDI spectra were obtained on a Bruker Microflex series MALDI-TOF using a matrix of dithranol saturated chloroform. IR spectra were recorded on a Perkin Elmer Spectrum 100 with a Universal ATR sampling accessory. Toluene was dried by passage through two columns of alumina and degassed by argon purge in a custom-built solvent purification system.

Molecular Controls for Tertiary Amine Functionalized PCBM, NDI, and DPP

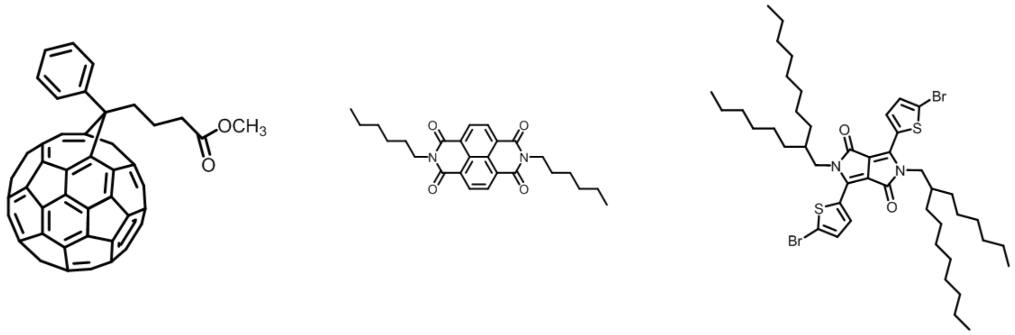

PCBM-Control **NDI-Control** **DPP-Control**

Thin film preparation

Thin films were cast from solution under mild heating [PDI-OH (water), cast at 75 ^{o}C; PDI-NMe$_2$, NDI-NMe$_2$, DPP-NMe$_2$ ($CHCl_3$), cast at 30 ^{o}C; PCBM-NMe$_2$ (methanol/toluene), cast at 30 ^{o}C]

Optical spectroscopy

Solution and thin film optical absorption spectra were collected using an Agilent Technologies Cary 60 UV-Vis spectrometer.

Continuous flow mass spectrometry

A Pfeiffer Vacuum Prisma quadrupole mass spectrometer was used to monitor composition upon dehydration of PDI-OH solution.

X-ray photoelectron Spectroscopy (XPS) analysis

XPS analysis was performed on a Kratos Axis Ultra DLD spectrometer (Kratos Analytical, Manchester, UK). All spectra were run using monochromated Al-k-alpha radiation; survey and high-resolution spectra were run at pass energies of 160 and 20 eV, respectively.

Electron paramagnetic resonance (EPR) analysis

PDI-OH and PDI-NMe$_2$.

Samples were prepared by dropcasting solutions onto 2 cm by 2 cm quartz microscope cover slides. The samples were cleaved into approximately 3 mm-wide pieces; the most uniform and rectangular pieces were saved as the final samples. Samples were then transferred into a dry glovebox with a nitrogen atmosphere. Before measurement PDI-OH samples were annealed for 0 hrs, 20min, 1hr, 4 hrs, and 16hrs

at 120 °C; PDI-NMe_2 and PDI-C6/PDI-NMe_2 composite samples were annealed for 4hrs. All samples were then inserted into 4 mm-diameter quartz EPR tubes. These tubes were sealed with plastic caps and Teflon tape inside the glovebox. They were then transferred outside of the glovebox and their EPR spectra were measured within two hours.

Spin concentrations were determined by comparing the integrated signal intensity of a sample with the integrated signal intensity of a standard of known concentration. In this experiment, the spin concentration calculations were complicated by the variation in film thickness that resulted from the dropcast. The thickness of each sample was measured using profilometry. The film was scraped off in three locations down to the substrate and the average difference in thickness across these sites was quoted as the average thickness of the dropcasted film. To calculate the spin concentrations, the normalized, integrated intensity of the samples was compared to the normalized, integrated intensity of a standard material. 2,2-Diphenyl-1-picrylhydrazyl (DPPH) was used as the standard sample and a calibrator for determining the spin concentrations of each material. All samples were measured in triplicate.

NDI-NMe_2, NDI-Control, DPP-NMe_2, and DPP-Control

EPR samples were prepared as discussed above and were all annealed for ~4hrs at 120 °C inside a glovebox prior to measurement.

PCBM-NMe_2 and PCBM-Control

For enhanced signal-to-noise, solutions were case and dried directly inside the quartz EPR tubes, which were then annealed for 4 hrs at 150 °C in a glovebox prior to measurement.

2. SYNTHESIS

Functionalized Perylene Diimide Derivatives

General procedure for the synthesis of dimethylamino perylene diimides

Perylene-3,4,9,10-tetracarboxylic dianhydride and the alkyl amine (4 equiv) were combined with imidazole in a round bottom flask equipped with a stir bar and sealed with a septum. The reaction vessel was purged with argon and subsequently heated with stirring at 130 °C for the indicated amount of time. At the conclusion of the reaction, the vessel was allowed to cool to room temperature, the contents were suspended in methanol, and the solid was collected by filtration using a 0.8 μm nylon membrane. The solid was washed with methanol and dried under vacuum to afford the pure product.

General procedure for the quaternization of dimethylamino perylene diimides

The dimethylamino substituted perylene diimide and iodomethane (4 equiv) were dissolved in chloroform in a round bottom flask equipped with a stir bar and condenser and refluxed for the indicated amount of time. At the conclusion of the reaction, the mixture was cooled to room temperature and filtered through a 0.8 μm nylon membrane. The solid was washed consecutively with chloroform, diethyl ether, hexane, and ethanol and dried under vacuum to afford the pure product.

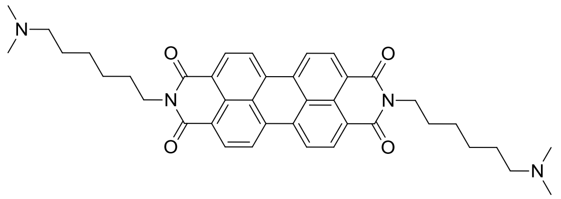

PDI-NMe$_2$

Bis(6-(dimethylamino)hexyl)perylene diimide

The general procedure was employed using perylene-3,4,9,10-tetracarboxylic dianhydride (1.042 g, 2.656 mmol), 6-(dimethylamino)hexylamine (1.522 g, 10.55 mmol), and imidazole (17.1 g, 251 mmol) in a 50 mL round bottom flask. After 1.5 h at 130 °C and workup, 1.64 g (96%) of the desired product was obtained. **^{1}H NMR** (600 MHz, $CDCl_3$): δ 1.37 – 1.55 (m, 12H), 1.77 (quin, *J* = 7.8 Hz, 4H), 2.21 (s, 12H), 2.26 (t, *J* = 7.5 Hz, 4H), 4.18 (t, *J* = 7.4 Hz, 4H), 8.41 (d, *J* = 8.0 Hz, 4H), 8.54 (d, *J* = 7.9 Hz, 4H). **^{13}C NMR** (125 MHz, $CDCl_3$): δ 27.30, 27.40, 27.83, 28.12, 40.71, 45.62, 59.97, 122.48, 122.96, 125.40, 128.64, 130.64, 133.52, and 162.76. **MS** (FD): m/z $[M]^+$ calcd for $[C_{40}H_{44}N_4O_4]^+$, 644.3; found, 644.3.

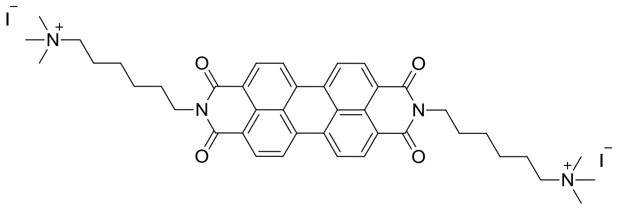

PDI-I

Bis(6-(trimethylammonium)hexyl)perylene diimide diiodide

The general procedure was employed using bis(6-dimethylamino)hexyl-perylene diimide (0.9086 g, 1.409 mmol), iodomethane (0.35 mL, 5.6 mmol), and chloroform (50 mL). After refluxing for 2 h and workup, 1.28 g (98%) of the desired product was obtained. **^{1}H NMR** (600 MHz, DMSO-d_6): δ 1.36 (p, *J* = 7.2 Hz, 4H), 1.45 (quin, *J* = 7.2 Hz, 4H), 1.67-1.75 (m, 8H), 3.04 (s, 18H), 3.26-3.31 (m, 4H), 4.08 (t, *J* = 7.5 Hz, 4H), 8.50 (d, *J* = 7.9 Hz, 4H), 8.83 (d, *J* = 7.9 Hz, 4H). **^{13}C NMR** (125 MHz, DMSO-*d6*): δ 22.08, 25.68, 26.18, 27.32, 52.14, 65.28, 121.75, 123.62, 124.46, 127.54, 130.19, 133.04, and 162.23. **MS** (ESI): m/z $[M - I]^+$ calcd for $[C_{42}H_{50}IN_4O_4]^+$, 801.2871; found, 801.2861.

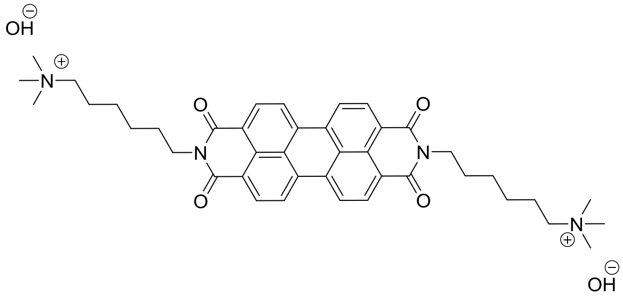

PDI-OH

PDI-OH preparation via counterion exchange

Synthesized PDI-I variants were dissolved in DI water at concentrations of ~2-3mg/ml and slowly eluted through a counterion exchange column (DOWEX 550A, Sigma). The resulting solution was deep purple in color and was used without further treatment.

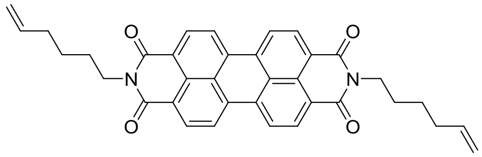

PDI-Alkene

N,N'–bis(hex-5-enyl)perylene-3,4,9,10-tetracarboxylic diimide

Perylene-3,4,9,10-tetracarboxylic dianhydride (610 mg, 1.55 mmol) and 1-amino-5-hexene[1] (661 mg, 6.66 mmol) were combined with imidazole (7.4 g) in a 50 mL round bottom flask equipped with a stir bar and sealed with a septum. The flask was purged with argon for approximately 5 min and heated with stirring in an oil bath maintained at 120 °C. After 2 h, the flask was cooled and the contents were suspended in 2M HCl and filtered using a 0.45 μm nylon membrane. The solid was washed with water, methanol, and acetone and dried under vacuum to yield 860 mg (quant) of the desired product as a red solid after drying under vacuum. **^{1}H NMR** (600 MHz, $CDCl_3$) δ: 1.56 (quin, *J* = 7.7 Hz, 4H), 1.79 (quin, *J* = 8.1 Hz, 4H), 2.16 (q, *J* = 7.2 Hz, 4H), 4.22 (t, *J* = 7.7 Hz, 4H), 4.93–5.07 (m, 4H), 5.83 (ddt, *J* = 17.0, 10.2, 6.7 Hz, 2H), 8.58–8.71 (m, 8H); **^{13}C NMR** (150 MHz, 1,1,2,2-tetrachloroethane-d_2) δ: 26.3, 27.5, 33.4, 40.4, 114.8, 120.2, 123.0, 123.1, 131.3, 134.4, 138.5, 163.2 ppm; IR (solid, cm^{-1}): 3071, 2932, 2860, 1692, 1651, 1593, 1579, 1439, 1340, 1250; **MS** (EI, m/z): calcd for $[C_{36}H_{30}N_2O_4]^+$ (M^+), 554.2206; found, 554.2195.

Tertiary Amine Functionalized C60

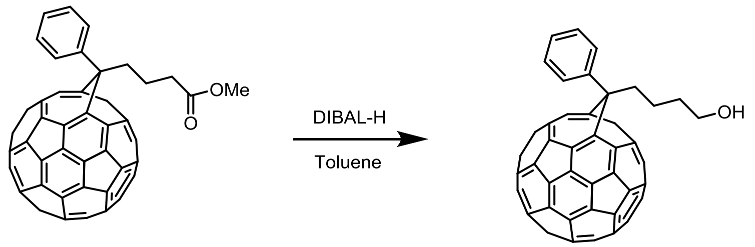

C60-OH

C_{60}-OH was synthesized according to literature methods by reduction of PCBM.[1] The product was purified using column chromatography on silica gel with 9:1 toluene to ethyl acetate as the eluent. C_{60}-OH was produced with a yield of 70%. **^{1}H NMR** (500 MHz, $CDCl_3$) δ (ppm): 7.93 (m, 2H), 7.55 (m, 2H), 7.48 (m, 1H), 3.74 (q, 2H), 2.90 (m, 2H), 1.92-1.98 (m, 2H), 1.78 (m, 2H); **MS** (MALDI-TOF, m/z): calcd for $[C_{71}H_{14}O]^+$ (M^+), 882.104; found, 883.035.

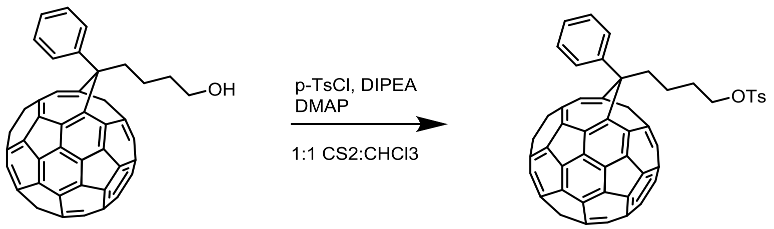

C60-OTs

Synthesis of C60-OTs. Under argon atmosphere, C_{60}-OH (0.068 mmol, 1 eq.), p-toluenesulfonic acid (0.272 mmol, 4 eq.), and DMAP (0.068 mmol, 1 eq.) were dissolved in chloroform (1.50 mL) and carbon disulfide (1.50 mL). DIPEA (0.08 mL, 7 eq.) was added via syringe. The reaction was allowed to stir for 15 hours. The reaction mixture was diluted with dichloromethane and washed three times with water. The organic layer was dried over sodium sulfate and the solvent removed under reduced pressure. The product was purified using column chromatography on silica gel starting with an eluent mixture of 2:1 hexane to chloroform and ending with a mixture of 1:2 hexane to chloroform. C_{60}-OTs was produced (0.042 g) with a yield of 60.%. **^{1}H NMR** (500 MHz, $CDCl_3$) δ (ppm): 7.87 (m, 2H), 7.78 (d, 2H), 7.55 (m, 2H), 7.48 (m, 1H), 7.34 (d, 2H), 4.12 (m, 2H), 2.83 (m, 2H), 2.45 (s, 3H), 1.85 (m, 4H). ; **^{13}CNMR** (600 MHz, $CDCl_3$) δ (ppm): 148.72, 147.71, 145.76, 145.17, 145.12, 145.02, 145.00, 144.77, 144.72, 144.64, 144.48, 144.38, 143.97, 143.73, 143.10, 143.00, 142.99, 142.91, 142.88, 142.22, 142.13, 142.09, 142.08, 140.98, 140.69, 137.97, 136.70, 133.11, 132.03, 129.87, 128.39, 128.22, 127.87, 79.82, 70.12, 51.85, 33.70, 28.83, 23.06, 21.63; **MS** (MALDI-TOF, m/z): calcd for $[C_{78}H_{20}O_3S]^+$ (M^+), 1036.113; found, 1036.286.

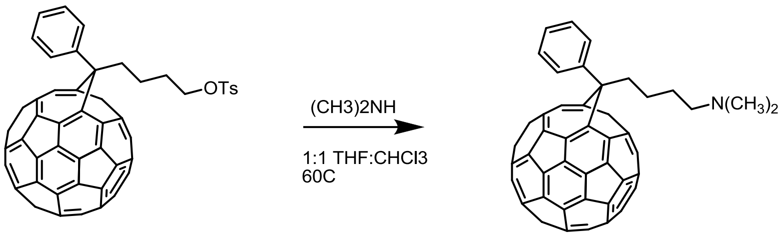

PCBM-NMe$_2$

Synthesis of PCBM-NMe$_2$. Under argon atmosphere, C_{60}-OTs (0.055 mmol, 1 eq.) was dissolved in THF (2 mL) and chloroform (2 mL). Dimethylamine (2 M in THF, 1.65 mmol, 30 eq.) was added via syringe. The reaction was heated to 60°C and allowed to stir for 20 hours. The reaction mixture was diluted with chloroform and washed three times with dilute aqueous potassium hydroxide. The organic layer was dried over sodium sulfate and the solvent removed under reduced pressure. The product was purified using column chromatography on silica gel starting with chloroform as the eluent and ending with chloroform plus 5% methanol. C_{60}-NMe$_2$ was produced (0.036 g) with a yield of 72%. **^{1}H NMR** (600 MHz, $CDCl_3$) δ (ppm): 7.90 (m, 2H), 7.54 (m, 2H), 7.46 (m, 1H), 2.88 (m, 2H), 2.48 (m, 2H), 2.34 (s, 6H), 1.85 (m, 2H), 1.73 (m, 2H). **^{13}C NMR** (600 MHz, $CDCl_3$) δ (ppm): 148.86, 147.92, 145.83, 145.15, 144.99, 144.76, 144.45, 144.38, 143.96, 143.74, 142.88, 142.08, 140.95, 140.69, 137.99, 132.09, 128.33, 80.06, 59.28, 45.03, 34.28, 24.79, 16.85. **MS** (MALDI-TOF, m/z): calcd for $[C_{73}H_{19}N]^+$ 909.152; found: 907.995.

Tertiary Amine Functionalized Naphthalene Diimide

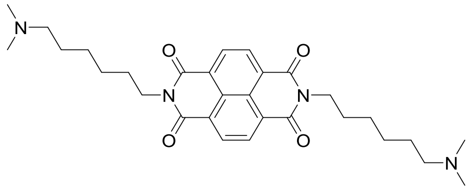

NDI- NMe$_2$

N,N'-bis(6-dimethylamino)hexyl-1,4,5,8-naphthalene tetracarboxylic diimide

1,4,5,8-Naphthalenetetracarboxylic dianhydride (3.73 mmol, 1 eq.) was suspended in anhydrous dimethyl acetamide (15 mL) and the contents were sparged with nitrogen for 15 min. N,N-dimethylethylenediamine (9.3 mmol, 2.5 eq.) was added via a syringe and the reaction mixture was stirred at 80˚C for 15 h. Upon cooling, the crude reaction mixture was poured into hexanes/isopropyl alcohol mixture (200 mL, 1:1) cooled to 0˚C. The resulting suspension was stirred and allowed to warm to room temperature. The solids were collected via filtration and subsequently washed with copious amounts of water. The product was purified over a short silica gel plug with ethyl acetate as the eluent. The solution was concentrated in vacuo to yield a beige solid (1.0 g, 65% yield). **^{1}H NMR** (600 MHz, $CDCl_3$) δ (ppm): 1.49-1.39 (m, 8H), 1.65 (quin, J = 7.5 Hz, 4H), 1.76 (quin, J = 7.2 Hz, 4H), 2.44 (s, 12H), 2.54 (t, J = 6.8 Hz, 4H), 4.19 (t, J

= 7.5 Hz, 4H), 8.73 (s, 4H); **^{13}C NMR** (150 MHz, $CDCl_3$) δ (ppm): 27.15, 27.27, 27.57, 28.15, 41.00, 45.48, 59.81, 126.75, 131.05, 162.93; **MS** (ESI): m/z calcd for $[C_{30}H_{40}N_4O_4H]^+$ ($M+H^+$), 521.30; found, 521.31.

Tertiary Amine Functionalized Diketopyrrolopyrrole[2,3,4]

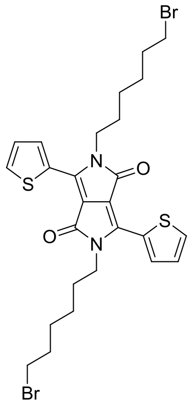

2,5-bis(6-bromohexyl)-3,6-di(thiophen-2-yl)-2,5-dihydropyrrolo[3,4-c]pyrrole-1,4-dione
3,6-Di(thien-2-yl)pyrrolo[3,4-c]pyrrole-1,4(2H,5H)-dione (534 mg, 1.78 mmol) was dissolved in DMF (20 mL) in a 100 mL round bottom flask equipped with a stir bar and sealed with a septum. The solution was cooled to 0 °C in an ice bath and sodium hydride (137 mg, 5.71 mmol) was added portionwise. The reaction was stirred for 10 minutes followed by the addition of 1,6-dibromohexane (1.64 mL, 10.7 mmol) via syringe. The reaction was allowed to warm slowly to room temperature and was stirred overnight. The reaction was diluted with water (100 mL) and extracted with dichloromethane (3 x 100 mL). The combined organic fractions were dried over $MgSO_4$, filtered through a short plug of celite, and concentrated with silica gel. The crude mixture was purified by column chromatography, eluting with a gradient of 50–100% dichloromethane in hexanes. The product was precipitated with methanol and collected by filtration to yield a dark purple solid after drying under vacuum (329 mg, 29%). **^{1}H NMR** (600 MHz, $CDCl_3$) δ: 1.41 – 1.55 (m, 8H), 1.77 (quin, J = 7.7 Hz, 4H), 1.87 (quin, J = 6.9 Hz, 4H), 3.40 (t, J = 6.8 Hz, 4H), 4.09 (t, J = 7.7 Hz, 4H), 7.30 (dd, J = 5.0, 3.9 Hz, 2H), 7.65 (dd, J = 5.0, 1.1 Hz, 2H), 8.93 (dd, J = 3.9, 1.2 Hz, 2H); **^{13}C NMR** (100 MHz, $CDCl_3$) δ: 26.2, 27.9, 29.9, 32.7, 33.8, 42.1, 107.8, 128.8, 129.8, 130.9, 135.5, 140.1, 161.5 ppm; IR (solid, cm^{-1}): 3109, 2925, 2843, 1658, 1558, 1403, 1225, 1105, 731, 706; **MS** (EI, *m*/*z*): calcd for $[C_{26}H_{30}Br_2N_2O_2S_2]^+$ (M^+), 624.0115; found, 624.0114.

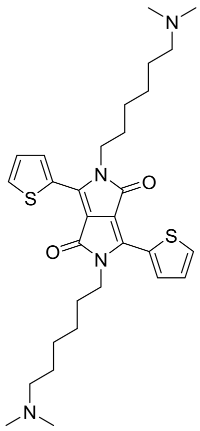

DPP-NMe_2

2,5-bis(6-(dimethylamino)hexyl)-3,6-di(thiophen-2-yl)-2,5-dihydropyrrolo[3,4-c]pyrrole-1,4-dione 2,5-Bis(6-bromohexyl)-3,6-di(thiophen-2-yl)-2,5-dihydropyrrolo[3,4-c]pyrrole-1,4-dione (143 mg, 0.228 mmol) was added to a 50 mL round bottom flask equipped with a stir bar and a septum. A 2 M solution of dimethylamine in THF (11 mL) was added via syringe and the mixture was stirred at room temperature. After 3 days, the tetrahydrofuran was removed under vacuum, the contents were dissolved in dichloromethane, and the solution was washed with water (1 x 100 mL) and 10% $NaHCO_3$ (1 x 100 mL). The organic fraction was dried over $MgSO_4$, filtered, and concentrated. After precipitation with cold hexanes, the product was isolated by filtration as a dark purple solid after drying under vacuum (84 mg, 67%). **^{1}H NMR** (600 MHz, $CDCl_3$) δ: 1.32 – 1.39 (m, 4H), 1.40 – 1.50 (m, 8H), 1.75 (quin, *J* = 7.6 Hz, 4H), 2.19 (s, 12H), 2.22 (t, *J* = 7.6 Hz, 4H), 4.07 (t, *J* = 8.0 Hz, 4H), 7.28 (dd, *J* = 5.0, 3.9 Hz, 2H), 7.64 (dd, *J* = 5.0, 1.2 Hz, 2H), 8.92 (dd, *J* = 3.9, 1.2 Hz, 2H); **^{13}C NMR** (100 MHz, $CDCl_3$) δ: 27.0, 27.3, 27.8, 30.1, 42.3, 45.6, 59.9, 107.8, 128.8, 129.9, 130.8, 135.4, 140.1, 161.5 ppm; IR (solid, cm^{-1}): 3091, 3065, 2925, 2852, 2760, 1651, 1557, 1224, 855, 697; **MS** (ESI, *m/z*): calcd for $[C_{30}H_{43}N_4O_2S_2]^+$ $(M+H)^+$, 555.2827; found, 555.2812.

3. FIGURES AND TABLES

(a)

Distribution Nitrogen XPS Signal

	N@399.6 eV (%)	N@402.8 eV (%)	N@400.3 eV (%)	
**Sample**	**[NMe_2]**	**[NMe_3^+]**	**[Imide N]**	**Tertiary amine signal (%)**
**PDI-NMe_2**	40.3	6.7	53.1	85.7
**PDI-OH As Cast**	7.7	42.1	50.2	15.5
**PDI-OH 20 Min**	26.3	19.6	54.1	57.2
**PDI-OH 1 Hour**	27.5	17.8	54.7	60.7
**PDI-OH 4 Hour**	29.4	15.6	55.0	65.4
**PDI-OH 16 Hour**	32.3	14.1	53.6	69.7

Tertiary amine signal (%):

$$\frac{[NMe_2]}{[NMe_2]+[NMe_3^+]}$$

Quaternary ammonium signal (%):

$$\frac{[NMe_3^+]}{[NMe_2]+[NMe_3^+]}$$

(b)

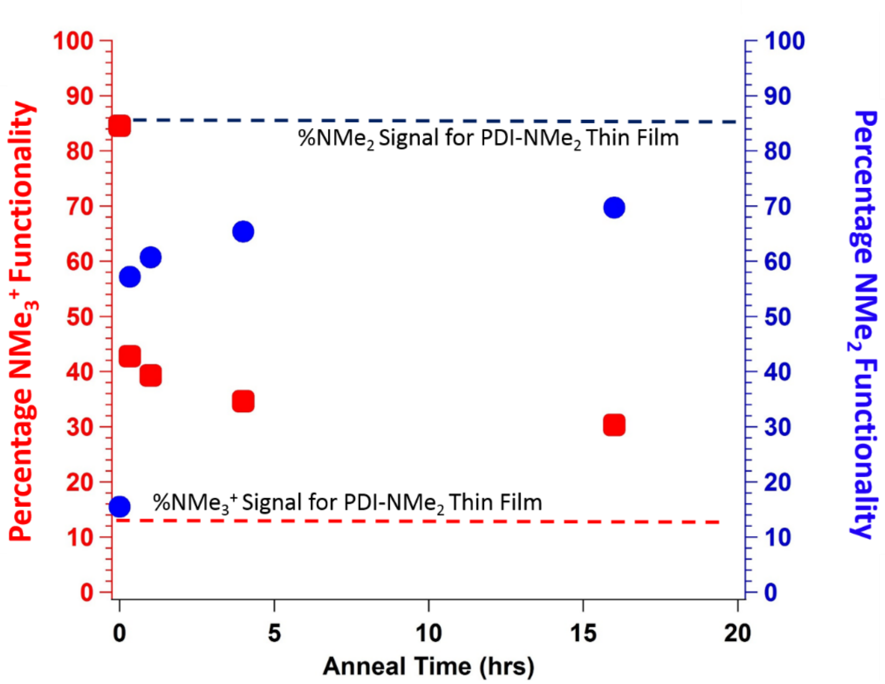

Figure S1: X-ray photoelectron spectroscopy analysis. (a) Comparison of the XPS nitrogen signal distribution between quaternary ammonium, imide, and tertiary amine environments for PDI-NMe_2 and PDI-OH (annealed for 0 hrs, 20min, 1hr, 4 hrs, and 16hrs) is shown. (b) Evolution of the fractional ammonium signal (in red) and the resulting tertiary amine signal in PDI-OH with annealing. The dashed lines represents the fractional tertiary amine signal (blue) and fractional ammonium signal (red) measured for PDI-NMe_2 in XPS. Presence of ammonium signal in annealed PDI-NMe_2 thin films may arise from radical cations formed upon charge donation from the tertiary amine groups. Radical cations along with unreacted quaternary ammonium functional groups are likely compounded in the remaining ammonium signal observed in PDI-OH thin films with extended annealing.

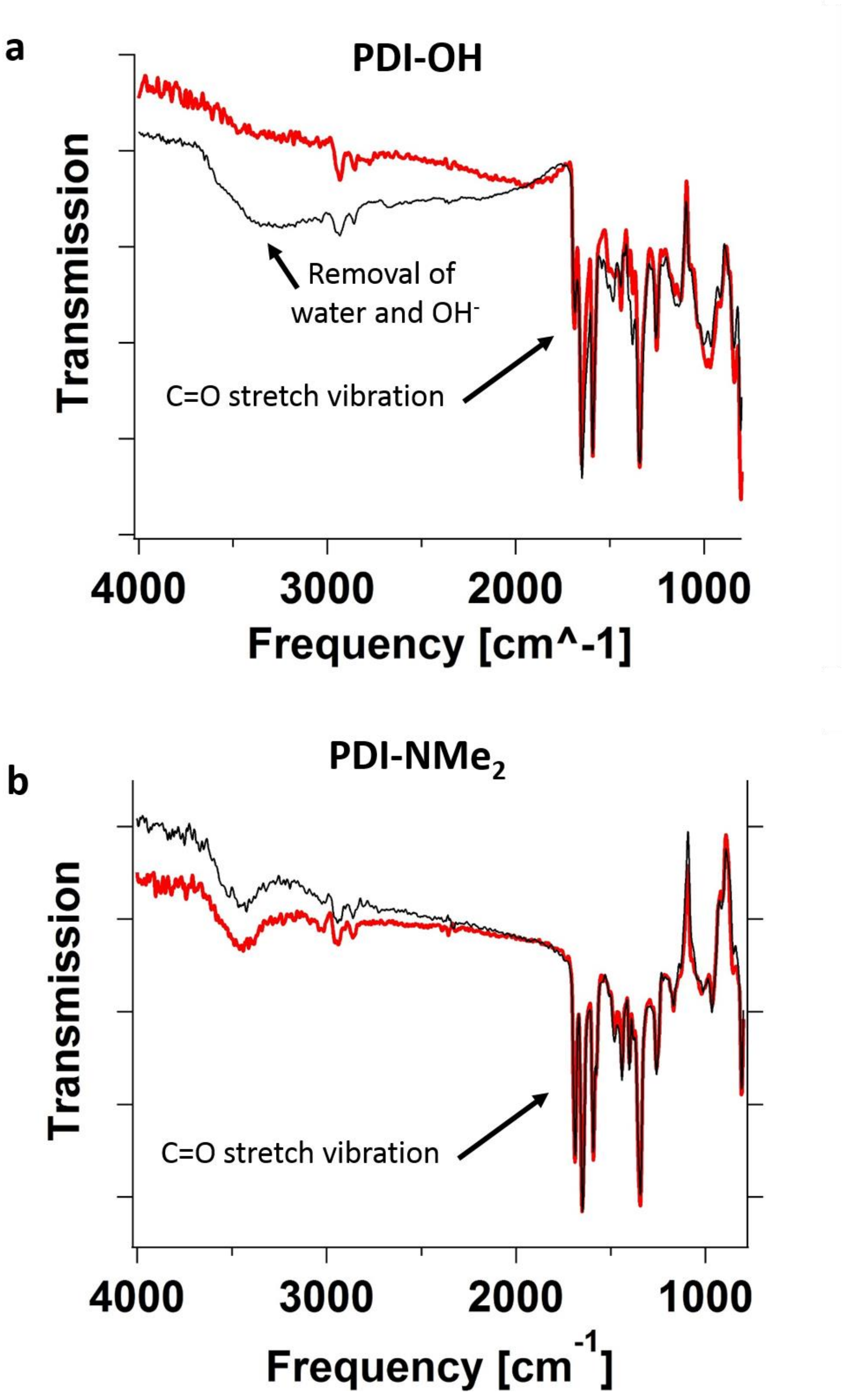

Figure S2: FTIR of (a) PDI-OH, (b) PDI-NMe_2. Comparison of as-cast (black line) and annealed (16hrs, 120 ^{o}C; red line) samples.

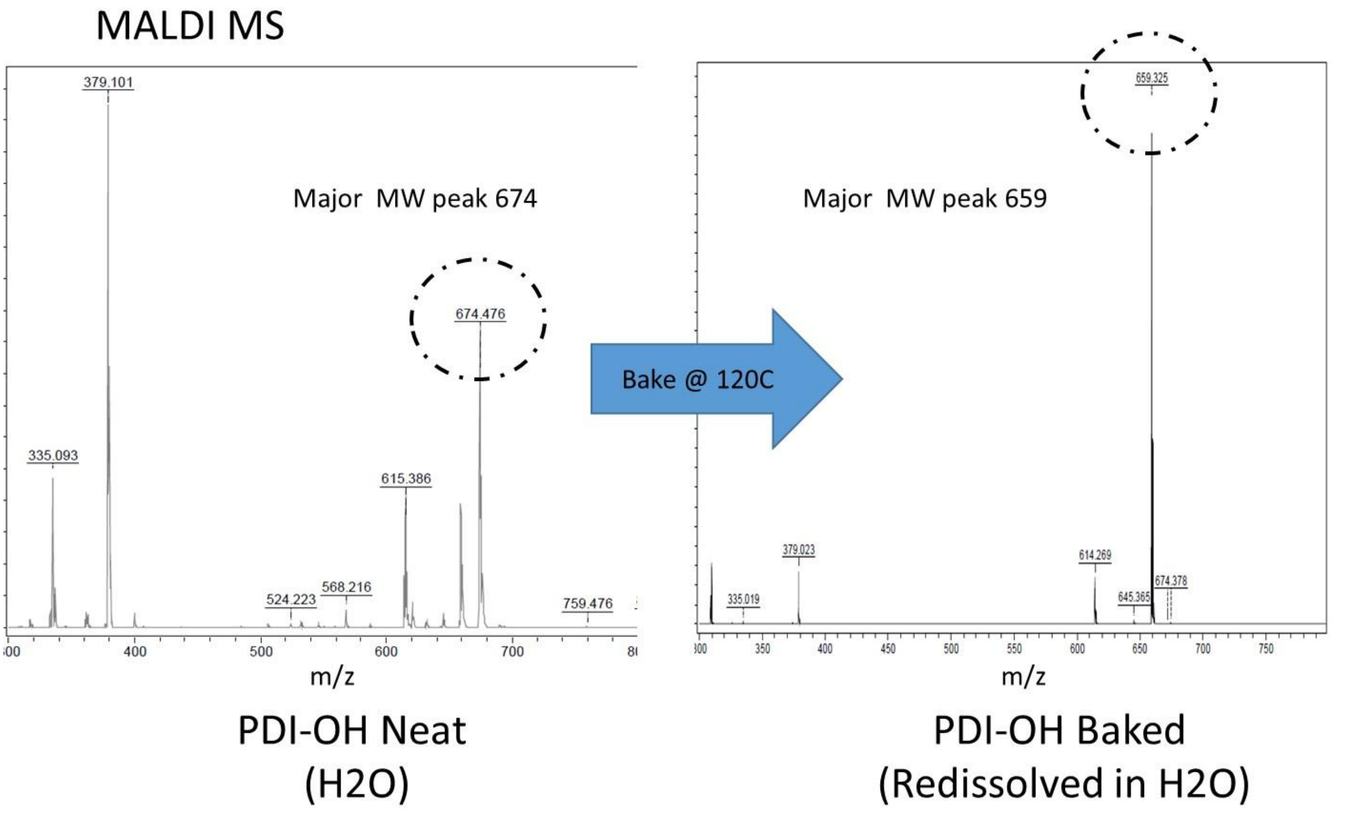

Figure S3: MALDI-MS of PDI-OH. Comparison of PDI-OH in its initial state and post annealing (120 ^{o}C, 16hrs) demonstrates the loss of a methyl group.

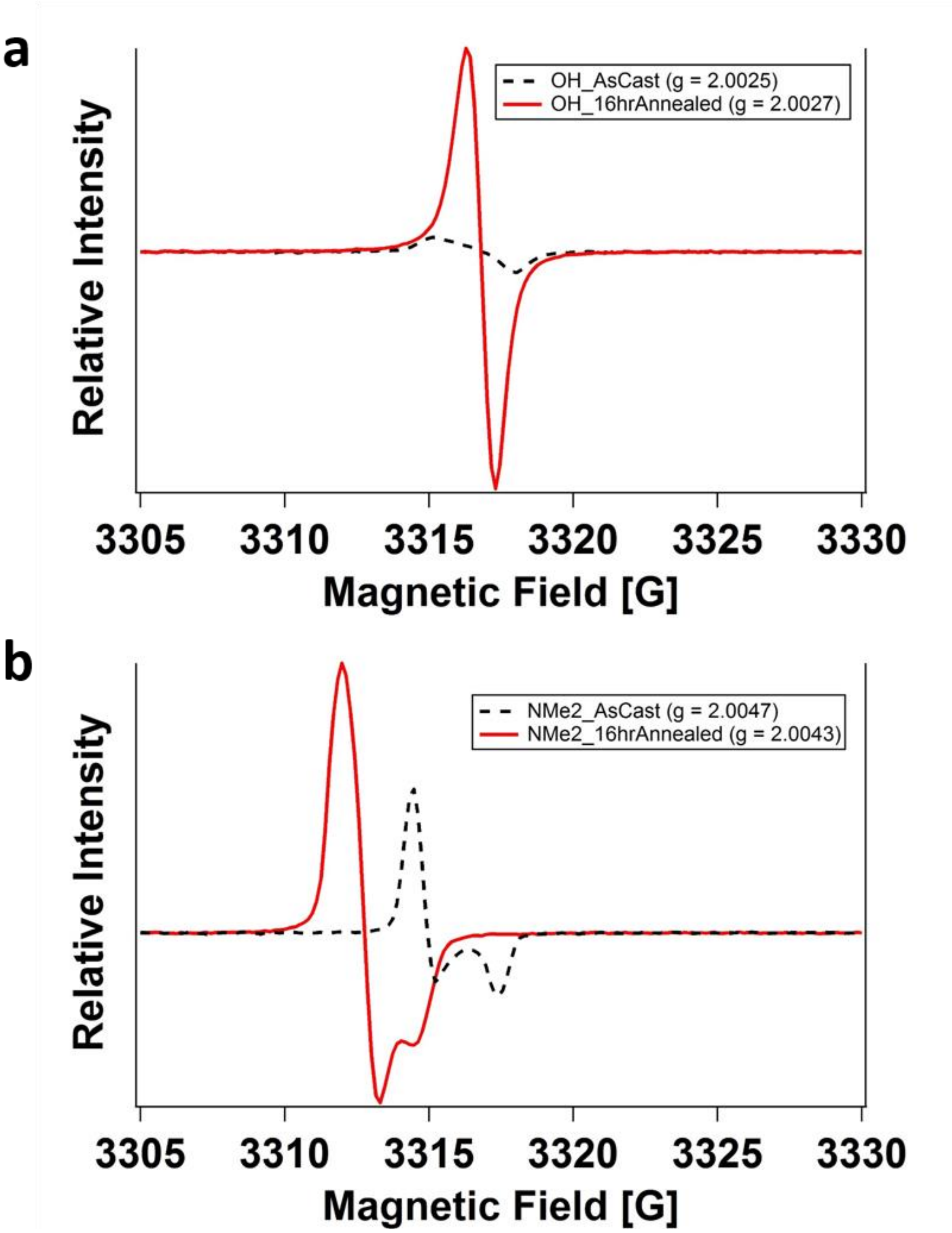

Figure S4: EPR raw spectra of as-cast and annealed (a) PDI-OH and (b) PDI-NMe_2. Asymmetry in the PDI-NMe_2 spectra is attributed to spin anisotropy within the sample in solid-state.

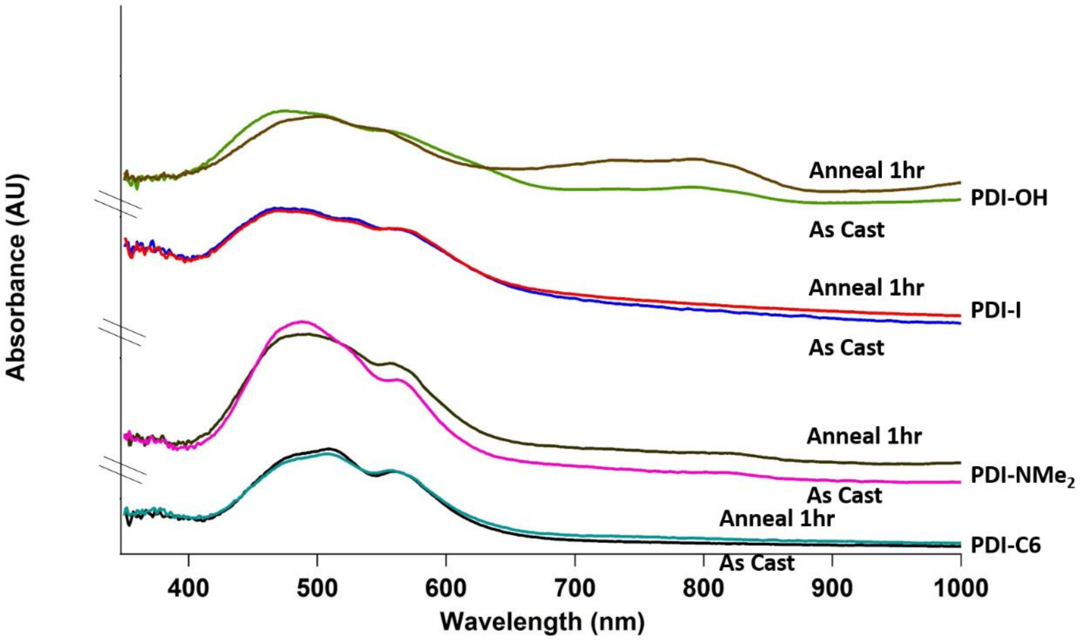

Figure S5: UV/VIS/NIR in solid-state. Comparison of the solid-state spectra for PDI-OH, PDI-I, PDI-NMe_2, and PDI-C6 in the as-cast state and the annealed states. Doping is observed in PDI-OH and PDI-NMe_2 upon film casting. The signal intensity in the PDI polaron region (700-1000nm) increases for PDI-OH and PDI-NMe_2 spectra with annealing, but remains unchanged for PDI-I and PDI-C6 with annealing.

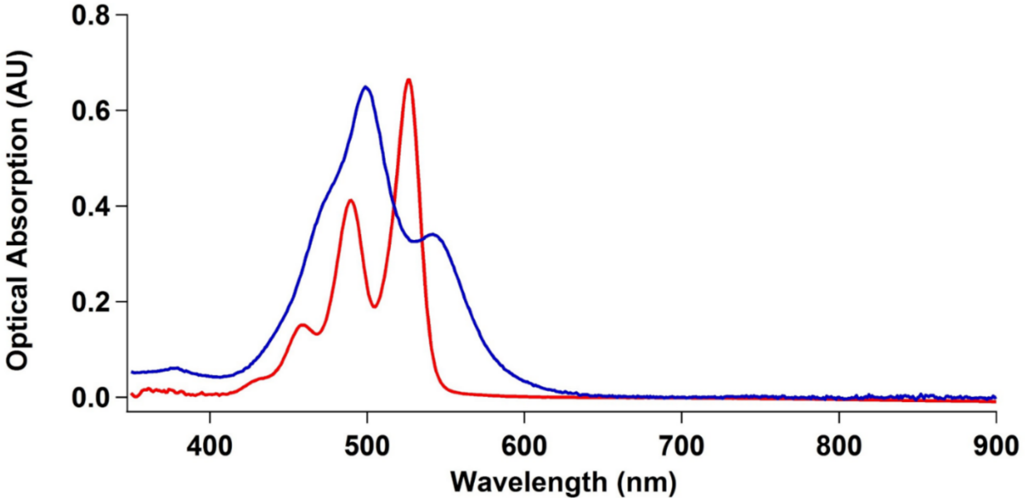

Figure S6: UV/VIS/NIR in Solution. Comparison of solution spectra for PDI-NMe_2 (in chloroform, red) and PDI-OH (in water, blue).

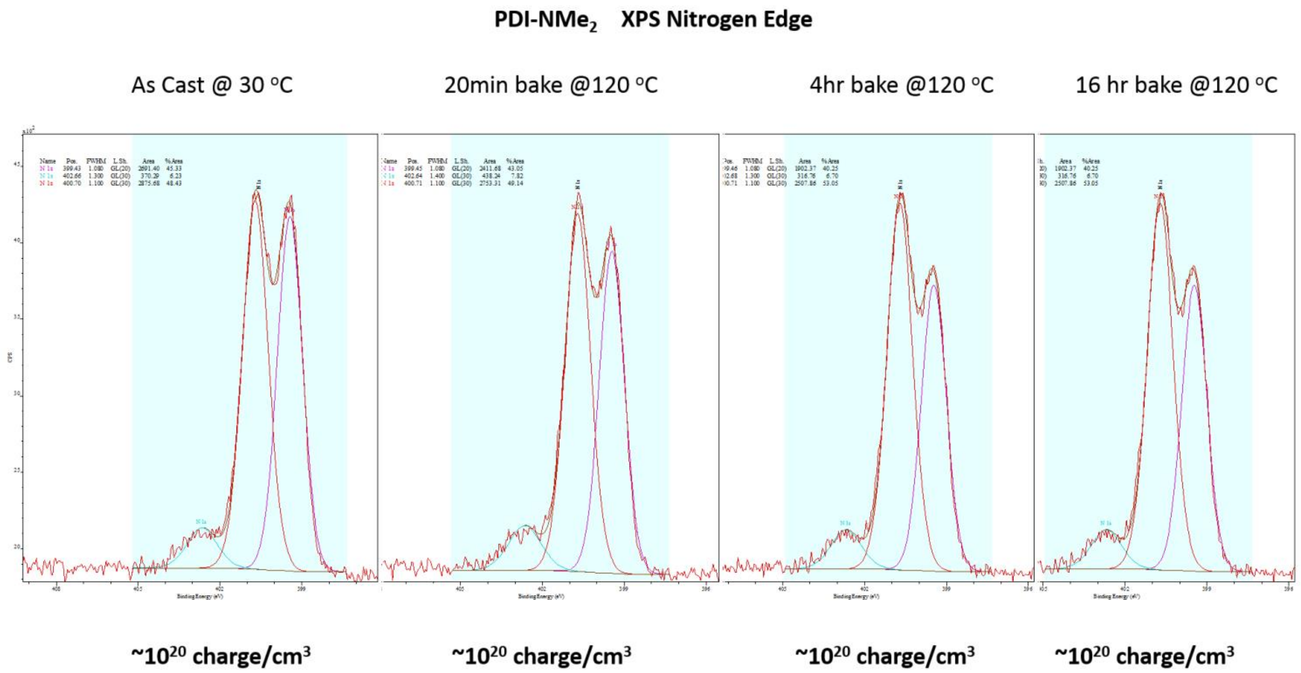

Figure S7: XPS comparison (Nitrogen edge) of PDI-NMe_2 after varied annealing times (as cast, 20 minutes, 4 hours, and 16 hours at 120 ^oC). Charge carrier concentrations were quantified with EPR for corresponding samples.

Estimation of Charge-Transfer Energetics in Self-Doping PDI Solid-State Thin Films

To estimate the charge transfer energetics associated with self-doping PDIs in the solid-state, we consider an alkylamine as a representative Donor (D) and the perylene dimide core as the Acceptor (A), which is shown in Figure S4. For the charge transfer to happen spontaneously, as is observed experimentally, the overall free energy change (ΔG_{CT}) must be negative.

We can determine ΔG_{CT} as $IP_D - EA_A - E_C$, where IP_D is the solid state ionization potential of the donor and EA_A is the solid state electron affinity of the acceptor, and E_C is the Coulombic stabilization energy. We use reported solid-state values for the electron affinity of perylene diimide (3.9 eV)[5] and ionization potential of alkylamines (6.5 eV)[6].

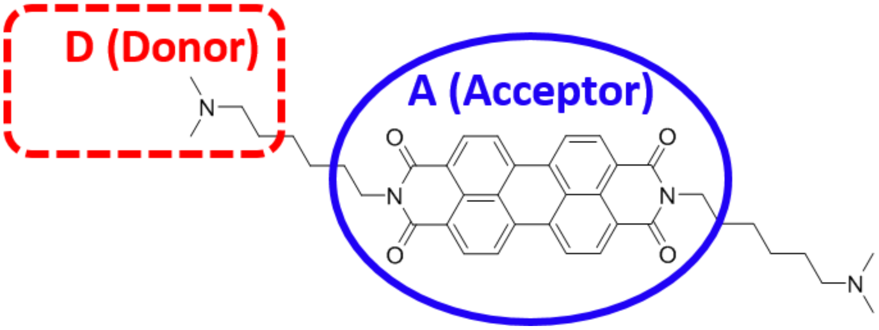

$A + e^- \rightarrow A^-$ **EA_A = ~3.9 eV**

$D \rightarrow D^+ + e^-$ **IP_D = ~ 6.5 eV**

Figure S8: (a) Self-doping PDIs are composed of perylene diimide core acceptors and tethered tertiary amine donors. To get a sense for the energetics involved in charge transfer, we consider, (b) trimethylamine as the model Donor and (c) perylene diimide as the Acceptor.

In solid-state, energetic stabilization effects, including electronic polarization[7] and Coulombic stabilization, aid in reducing the energy barrier. Polarization effects are already taken into account when using solid state EA and IP values.

The Coulombic stabilization can be roughly approximated as, $\mathbf{C} = -\frac{e^2}{4\pi\epsilon_o \epsilon * d}$, where $\frac{\mathbf{1}}{\mathbf{4\pi\epsilon_o}}$ = 8.98755E9 Nm^2/C^2; **e** is the charge constant (1.6E-19 C); ϵ is the dielectric constant (~3 for organic solid film). If the separation distance between D^+ and A^-, **d**, is taken to be ~4 $\dot{A}$, then,

$$\mathbf{C} = -\frac{e^2}{4\pi\epsilon_o \epsilon * d} = \mathbf{\sim 1.2\ eV}$$

Therefore, even with the aid of Coulombic stabilization, we estimate ΔG_{CT} ~ +1.4 eV. This energy barrier is well beyond the ~25-40 meV of thermal excitation that would be present at the temperatures considered in this study.

While the doping effect were observed even when processing films in the dark, it is possible that rapid visible light photo-induced excitation during sample transfer was sufficient to aid the sample doping.

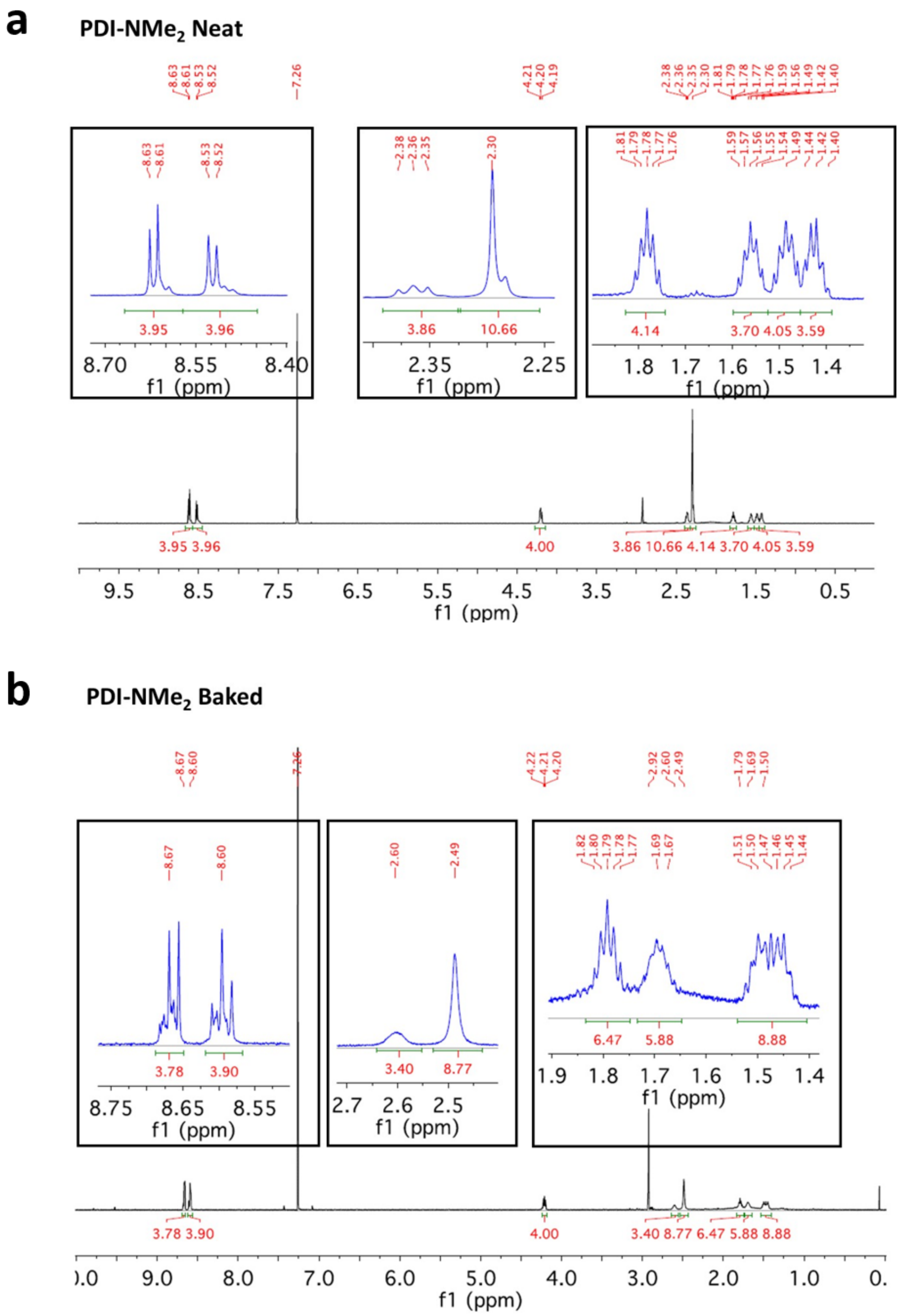

Figure S9: ^{1}H NMR spectra recorded in $CDCl_3$ (500 MHz). Comparison of PDI-NMe_2 neat and post annealing (120 ^{o}C, 16hrs) suggests that longterm thermal treatment results in chemical transformation of PDI-NMe_2.

Estimation of LUMO Levels for Various Molecular Cores

Molecular Core	LUMO (Estimated)
PDI	3.9 eV [5]
PCBM	4.1 eV [8]
NDI	3.9 eV [5]
DPP	3.5 eV [9]

Figure S10: LUMO levels for PDI, PCBM, NDI, and DPP molecular systems.

4. REFERENCES

1. W-B. Zhang, J. He, X. Dong, C-L. Wang, H. Li, F. Teng, X. Li, C. Wesdemiotis, R. Quirk, S. Cheng, *Polymer* 2011, **52**, 4221-4226.

2. Z. K. M. Abd El Samii, M. I. Al Ashmawy, J. M. Mellor, *J. Chem. Soc. Perkin Trans. 1* 1988, 2517–2522.

3. C. H. Woo, P. M. Beaujuge, T. W. Holcombe, O. P. Lee, J. M. J. Fréchet, *J. Am. Chem. Soc.* 2010, **132**, 15547–15549.

4. C. Papageorgiou, X. Borer, *Helvetica Chimica Acta*, 1988, **71**, 1079–1083.

5. X. W. Zhan, A. Facchetti, S. Barlow, T. J. Marks, M. A. Ratner, M. R. Wasielewski, S. R. Marder, *Adv. Mater*. 2011, **23**, 268.

6. Y. H. Zhou, C. Fuentes-Hernandez, J. Shim, J. Meyer, A. J. Giordano, H. Li, P. Winget, T. Papadopoulos, H. Cheun, J. Kim, M. Fenoll, A. Dindar, W. Haske, E. Najafabadi, T. M. Khan, H. Sojoudi, S. Barlow, S. Graham, J. L. Bredas, S. R. Marder, A. Kahn and B. Kippelen, *Science*, 2012, **336**, 327-332.

7. G. Hill, A. Kahn, Z. G. Soos, J. R. A. Pascal, *Chem. Phys. Lett.* 2000, **327**, 181-188.

8. D. Muhlbacher, M. Scharber, M. Morana, Z. G. Zhu, D. Waller, R. Gaudiana, C. Brabec, *Adv Mater*, 2006, **18**, 2931.

9. P. Sonar, G. M. Ng, T. T. Lin, A. Dodabalapur, Z. K. Chen, *J Mater Chem* 2010, **20**, 3626.
